# Nicotine or marijuana vaping exposure during pregnancy and altered immune responses in offspring

**DOI:** 10.20517/jeea.2024.03

**Published:** 2024-04-15

**Authors:** Jonas M. Ndeke, James E. Klaunig, Sarah Commodore

**Affiliations:** 1 Department of Epidemiology and Biostatistics, Indiana University School of Public Health, Bloomington, IN 47405, USA.; 2 Department of Environmental and Occupational Health, Indiana University School of Public Health, Bloomington, IN 47408, USA.

**Keywords:** Cancer risk, pregnancy, offspring, electronic nicotine delivery systems, e-cigarette, marijuana vaping

## Abstract

Electronic nicotine delivery systems (ENDS) - which include electronic cigarettes or e-cigarettes, or simply e-cigs, and marijuana vaping have become increasingly popular. ENDS devices have been established as one of the tobacco quit methods and promoted to be safer compared to traditional tobacco cigarettes. Emerging evidence demonstrates that e-cigarette and marijuana vape use can be harmful, with potential associations with cancer. Herein, we summarize the level of evidence to date for altered immune response, with a focus on cancer risks in the offspring after maternal use of, or aerosol exposures from, ENDS or marijuana vape during pregnancy. From 27 published articles retrieved from PubMed, we sought to find out identified carcinogens in ENDS aerosols and marijuana vapor, which cross the placental barrier and can increase cancer risk in the offspring. Carcinogens in vaping aerosols include aldehydes, metals, tobacco-specific nitrosamines, tobacco alkaloids, polycyclic aromatic hydrocarbons, and volatile organic compounds. Additionally, there was only one passive vaping exposure case study on a human fetus, which noted that glycerol, aluminum, chromium, nickel, copper, zinc, selenium, and lead crossed from the mother to the offspring’s cord blood. The carcinogens (metals) in that study were at lower concentrations compared to the mother’s biological matrices. Lastly, we observed that *in utero* exposures to ENDS-associated chemicals can occur in vital organs such as the lungs, kidneys, brain, bladder, and heart. Any resulting DNA damage increases the risk of tumorigenesis. Future epidemiological studies are needed to examine the effects of passive aerosol exposures from existing and emerging electronic nicotine and marijuana products on developing offspring to cancer.

## INTRODUCTION

Since the landmark 1964 report by the United States Surgeon General^[1]^, tobacco smoking has been established to contain more than 70 carcinogens, which are associated with multiple cancers, primarily lung cancer^[2]^. Efforts for lung cancer prevention have used various tobacco cessation approaches such as nicotine patches, lozenges, and gum. However, since 2006, electronic nicotine delivery systems (ENDS) have become a popular alternative for nicotine replacement therapy. ENDS products encompass a variety of devices, including vape pens, pods, mods, and electronic cigarettes, commonly referred to as e-cigarettes. E-cigarettes and other ENDS have been promoted as safer alternatives compared to traditional tobacco cigarettes^[3–6]^, including by official health agencies such as the Food and Drug Administration in the United States^[7]^, the National Institute for Health and Care Excellence (NICE) in the United Kingdom^[8]^, and the Ministry of Health in New Zealand, which deems vaping as not harmless but much less harmful than smoking and susceptible to help some people quit smoking^[9]^. This argument has served as the basis for its promotion and marketing messaging, which has been especially successful among teenagers^[10–13]^ and young adults^[14]^, as well as in pregnant women^[15–17]^. Along with ENDS, marijuana vaping has gained popularity, including in pregnant women^[18]^. However, there is emerging evidence to demonstrate that both e-cigarette (which contains nicotine)^[19]^ and marijuana vape use can be harmful, with potential associations with cancer^[19]^, though there is a need for more studies in both animal models and humans to affirm or refute the currently available evidence and to bring new knowledge where evidence is not available yet. Especially in pregnant users, nicotine, cannabis products, and other chemicals present in either vaping device or the vapor itself can cross the placental barrier and expose the developing fetus to a novel chemical mixture whose long-term effects have not been characterized. Notably, though perceived by pregnant women who smoke as a good aid to quit smoking as in the general population^[17]^, both the World Health Organization and the Centers for Disease Control and Prevention recommend against the use of vaping during pregnancy^[20,21]^. It is important to explore the available knowledge on how active nicotine or marijuana vaping, or passive exposures to one or both of these vapors during pregnancy may present cancer risks to offspring.

The National Academies of Sciences, Engineering and Medicine’s committee on the review of the health effects of ENDS identified health consequences of e-cigarettes and listed a set of conclusions based on the cancer endpoint studied and the level of evidence provided. This committee then found no available evidence on whether or not e-cigarette use is associated with intermediate cancer endpoints in humans. The committee also found limited evidence from biomarker-based *in vivo* animal studies to support the hypothesis that long-term e-cigarette use could increase the risk of cancer and, finally, the committee found substantial evidence that some chemicals like formaldehyde present in e-cigarettes may cause DNA damage and mutagenesis. From this latter evidence, the committee developed a framework that describes different plausible pathways leading to cancer through the use of ENDS, while calling for stronger evidence in animal models and available evidence in humans^[19]^. Some of these pathways include DNA damage and mutagenicity, inflammation, reactive oxygen species, and other reactive intermediates. This framework explains the mechanisms and intermediate outcomes influencing cancer outcomes after exposure to ENDS aerosols^[19]^. One of the substantial pieces of evidence that indicate the health risk related to the use of ENDS in the framework is that among experienced adult ENDS users, nicotine intake can be comparable to that from combustible tobacco cigarettes. Nicotine contributes to smoking initiation and subsequent addiction, and exposes the user to cardiovascular events through atherosclerosis^[22–27]^, especially in persons with underlying coronary artery disease^[23,24]^. Moreover, preliminary findings show that nicotine, especially through its derivates when smoked or consumed orally, may contribute to cancer development by stimulating certain key processes^[28]^, including the activation of nicotine acetylcholine receptors. However, the cancer risk from nicotine is very low (at least to date)^[19]^, as the nicotine contained in ENDS aerosols does not carry enough tobacco-specific nitrosamines or other pyrolysis products to cause cancer. The evidence for cancer risk from ENDS use may be important in relation to flavorants and humectants, which are present in the ENDS aerosols resulting from the heating and aerosolization of the e-liquid^[29]^. Furthermore, ENDS aerosol samples showed the presence of compounds such as arsenic, manganese, nickel, chrome, and lead^[30]^, while acrolein, methacrolein, tetrahydrocannabinol (THC), benzene, Vitamin E acetate, and terpenes were found in vaping cannabis mixtures^[31]^. Additionally, formaldehyde can be formed when the glycerol or propylene glycol in ENDS products are heated^[32]^; it was also found in 4/16 e-liquids assessed by a 2022 study^[33]^. These are all well-established carcinogens^[6,19,34]^, though for THC, only limited evidence of a statistical association between current, frequent, or chronic cannabis smoking and non-seminoma-type testicular germ cell tumors has been conclusive^[35]^ and more evidence is needed. Technology-wise, newer generation ENDS products have been associated with an increased immunosuppression^[36,37]^, which is a risk factor for cancer^[38–40]^. Likewise, newer versions of marijuana vaping come with a wide variety of chemicals, with both known and unknown cancer risks^[41]^. It is important to note that the experiment conducted by Tang *et al.* may not be representative of the aerosol chemistry of cannabis vapor products by pregnant individuals or those in their households^[31]^. However, long-term use of nicotine and/or marijuana vaping needs to be addressed for cancer risk assessment and prevention based on available plausible biological pathways to cancer development. Stronger evidence, however, will require many years of research on long-term cancer outcomes in the general population^[28]^.

Given the still building evidence on the carcinogenicity of ENDS and marijuana vaping, no study on humans has been reported on the long-term cancer outcomes on children born from mothers who smoked or were exposed to either nicotine or marijuana vaping, or both, during pregnancy. Nevertheless, for the established carcinogens known to cross the placental barrier, there is a need to assess cancer risk in the offspring, using the same conceptual framework presented by the National Academies’ review committee^[19]^. To this end, we aimed to summarize the level of evidence to date for altered immune responses with a special focus on cancer risks in the offspring resulting from vaping nicotine or marijuana, or both, during pregnancy. Specifically, we conducted a literature review using the PubMed database. To assist with our review, we sought to answer the following questions:

1. What are the currently identified carcinogens contained in the nicotine and marijuana aerosols and devices, respectively?
2. Which of these carcinogens are known to cross the placental barrier, and at what proportion compared to their respective concentrations in the mother’s biological samples?
3. Are these carcinogens associated with (future) risk of cancer in the offspring?

## METHODS

### Search strategy

We searched PubMed for relevant studies published between 2003 (the year the first electronic cigarettes were marketed) and December 2022. Our initial search string combined key words related to (1) the compounds identified in ENDS; (2) ENDS; and (3) pregnancy. However, this strategy yielded no study and we needed to combine results from separate component search strings, which are well detailed in the Supplementary Materials. Noteworthy, all component strings that combined “marijuana vaping”, or associated terms, with pregnancy yielded no studies. A possible explanation is the newness of this category of vaping devices and their still low inclusion in research involving pregnant women. Therefore, to capture the available data on the offspring’s prenatal exposure to marijuana compounds, we expanded our search to studies involving marijuana and pregnancy, without the search term “vaping”. However, such exposure should afterward be restricted to the only compounds identified in marijuana vape devices^[41]^. We also reviewed reference lists from the relevant studies yielded by this search, as well as studies obtained from colleagues and those from individual journals that had been previously identified by the team but not captured by this search strategy.

### Screening

**Figure 1.**
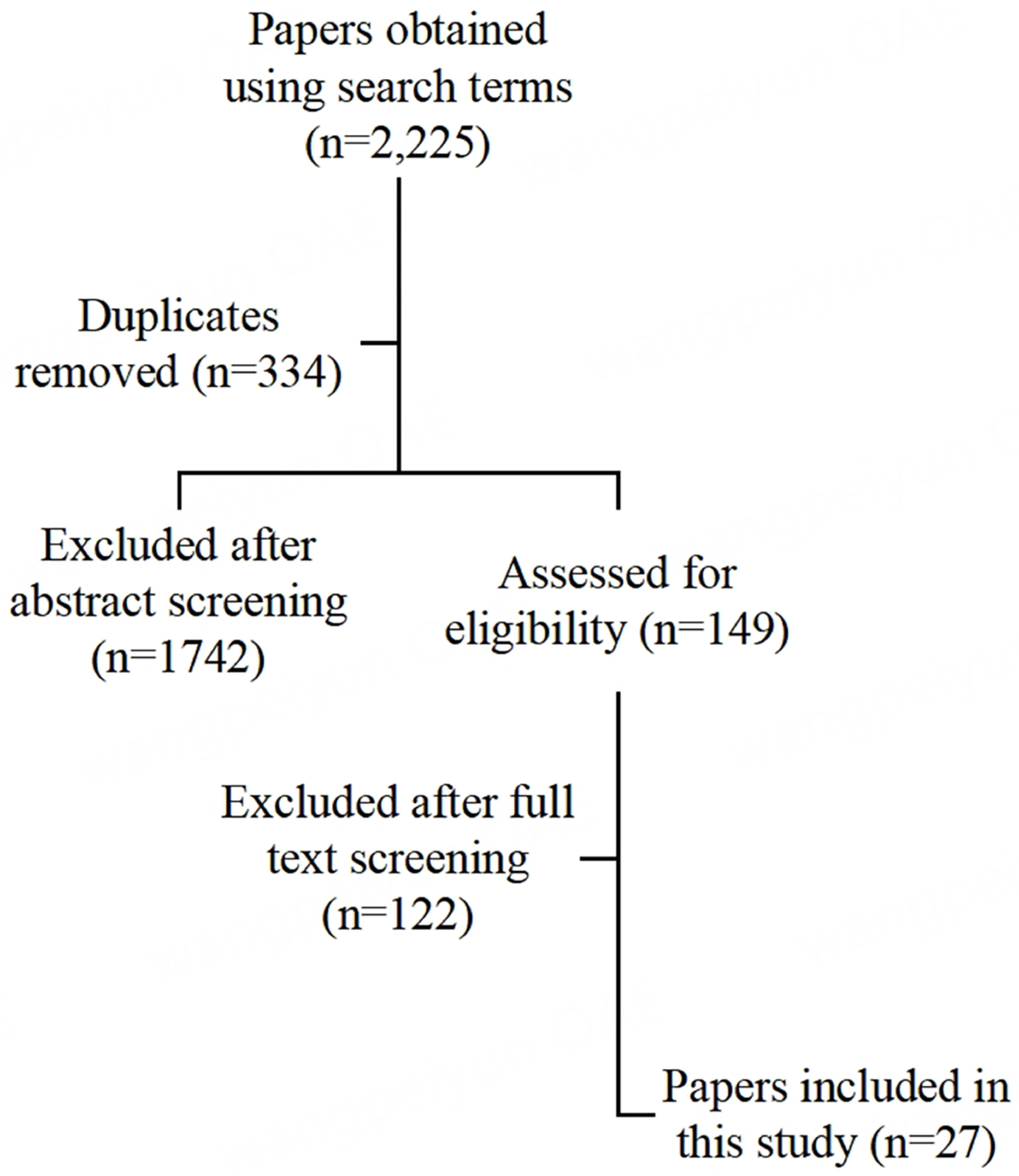
PRISMA diagram showing the number of papers sorted through each stage of screening for this review article.

We initially screened titles and abstracts and excluded irrelevant articles, then screened full articles for final inclusion. The full list of our full-paper inclusion and exclusion criteria is available in the Supplementary Materials. Among others, we excluded articles if: (1) they were commentaries, letters to the editor, editorials, qualitative studies, *etc.*; (2) they discussed ENDS- or marijuana-contained substances, but without their health consequences; (3) they were published before 2003 or after 2022. Each reviewer (JMN and SC) screened studies independently at both phases and the two reviewers resolved discrepancies together in discussions. Figure 1 shows the number of included and excluded papers.

## DISCUSSION

Herein, we conducted a narrative review on the potential for ENDS- and marijuana vaping-associated chemical exposures (either through active maternal nicotine or marijuana vape, or passive aerosols from another householder) to introduce carcinogenic compounds to and impact a developing fetus. We acknowledge that there are numerous other sources of environmental contaminants and the potential for transfer of organic contaminants (and/or their metabolites) to the developing fetus. However, this is beyond the scope of the current work, and herein, we only discuss ENDS- and marijuana vaping-associated chemical exposures.

Both nicotine and cannabis vapes contain complex mixtures of chemicals, and such complexity increases with thermal degradation chemistry^[42]^. The study by Li *et al.* underscores the need for further research on individual components to gain a thorough understanding of aerosol composition from using various vaping devices while accounting for individual user settings^[43]^.

It is important to acknowledge that a discussion on the measurement of metabolites or adducts of carcinogenic substances would be helpful, since some of these compounds require metabolic activation to exert adverse effects. Some birth cohort studies, even with low smoking prevalence, have shown that urinary biomarker values and self-reported smoking can be used to assess passive exposures^[44]^. Interestingly, one birth cohort study observed an association between offspring DNA methylation and metabolites of cigarette smoke (except for nicotine)^[45]^. Presumably, the toxic effects of prenatal nicotine exposure may be exerted by downstream metabolites, rather than nicotine. While cotinine and NNAL could be used as a measure of tobacco smoke exposure, cotinine may be a more precise candidate to detect passive exposures^[46]^. Metabolic intermediates of tobacco-specific nitrosamines such as 4-(methylnitrosamino)-1-(3-pyridyl)-1-butanol (NNAL) and its glucuronides, as well as 4-(methylnitrosamino)-1-(3-pyridyl)-1-butanone (NNK), are potent carcinogens^[47]^. Then, during further biotransformation, metabolic intermediates of NNAL and NNK such as methane diazonium ions can react with DNA to form methyl adducts, such as 7-methylguanine (m^7^Gua) (PMID: 10064856)^[48]^. m^7^Gua can be used as a marker of adverse health effects, and its concentrations have been found to be higher among subjects exposed to passive cigarettes and heated tobacco products^[46]^. Similar measurements, conducted from the urines of pregnant individuals who are exposed to ENDS and marijuana vape products, would provide a more thorough risk assessment and characterization.

Excessive oxidative stress, such as oxidative damage to DNA, cells, and other macromolecules, has been implicated in cancer initiation and promotion^[49,50]^. The subject of our focus in this review, the developing fetus, has few defense capabilities of protecting itself from enhanced oxidative stress and inflammation that may come from ENDS- or marijuana vapor-associated chemicals that enter the maternal-fetal circulation. For instance, fetuses have reduced antioxidants and limited activities of most antioxidative enzymes^[51]^ as their organs are still in the formation stages. As such, the fetus may be at increased risk for cancer initiation or promotion because of increased reactive oxygen and nitrogen signaling, oxidative DNA damage, or even alterations in its genomic structure introduced by these exogenous compounds through maternal or environmental nicotine or marijuana vape use. We postulate that prenatal programming of cancer may occur with exposures to ENDS- or vaping marijuana-associated compounds. Moreover, as has been proposed before with marijuana vape oil^[52]^ and with ENDS^[53]^, we hypothesize that certain types of cancer may be initiated in the developing fetus, due to the combination of low antioxidative activity and increased oxidative stress and inflammation. We provide a brief summary of the studies that informed our hypothesis [Tables 1 and 2]. For the rest of the discussion, we consider three main questions below, addressed separately regarding ENDS and marijuana vape devices.

### A look back to cancer risk in offspring of women who smoked traditional tobacco cigarette

Prior to directly addressing this question for ENDS and marijuana vaping, it is important to briefly look back to what studies found on the cancer risk in the offspring of women who smoked traditional tobacco cigarettes during pregnancy. Conflicting results emerged from this question over the last four decades, but there are converging findings on specific endpoints. First, DNA methylation has been demonstrated in this population of offspring^[78–80]^, involving pathways including those leading to cancer development. Second, studies including meta-analysis^[81]^ revealed that central nervous system cancers^[81]^, of which retinoblastoma^[82]^, were the ones consistently at an increased risk among children whose mothers smoked during pregnancy. However, one recent large meta-analysis^[83]^ did not find an increased risk of neuroblastoma in this population and recommended a longitudinal study. Third, studies involving pregnant mothers (and fathers in some) showed no or unclear association. Leukemia^[81,84–87]^ and lymphoma^[81,88]^ have shown conflicting findings within and between studies, while an increased risk of testicular cancer, although much researched, was not demonstrated over time^[89]^. This non-exhaustive list of studies indicates that, like tobacco smoking and despite the two types of smoking not exposing the fetus to the same types of carcinogens, our attention should be directed to the developing fetus when considering the possible cancer risk from maternal nicotine and marijuana vaping during pregnancy. As this is a developing topic, the target organs for cancer risk in the offspring may not be specified before the risk itself and its endpoints are well established.

### ENDS-contained carcinogens, their transmission from mother to fetus, at what proportion compared to their respective concentrations in the mother’s biological samples, and the associated altered fetal immune response

#### Harmful carcinogens and their transmission

A wide range of compounds are formed at different temperatures during ENDS use^[75]^. Hence, multiple chemicals and pathways may interact, leading to adverse health effects, since individual user settings may differ and the number of chemicals and/or byproducts is infinite. Before we examine some of the identified carcinogens in ENDS aerosols, it is important to understand the role played by actively and/or passively inhaling ENDS aerosols. Compounds identified in the heated ENDS aerosol exposure to e-cig aerosols lead to measurable oxidative and inflammatory responses in lung cells and tissues, which, in turn, may result in not fully studied health consequences including pathways to cancer initiation^[53]^. In terms of carcinogenic chemicals that pregnant ENDS users (and their developing offspring) may be exposed to, aldehydes, metals, tobacco-specific nitrosamines, tobacco alkaloids, polycyclic aromatic hydrocarbons, and volatile organic compounds may be familiar culprits^[90]^. It is known that hydrocarbons and reactive aldehydes are formed when the liquids in ENDS are heated to high temperatures around 500 °F^[91]^. Some of these compounds (some of which have been found in counterfeit e-cigarette cartridges such as isoprene, acetaldehyde, ethylbenzene, toluene, acrolein, 1,3-butadiene, and benzene) have been listed by the FDA as harmful or potentially harmful constituents in tobacco products and tobacco smoke^[92]^.

**Figure 2.**
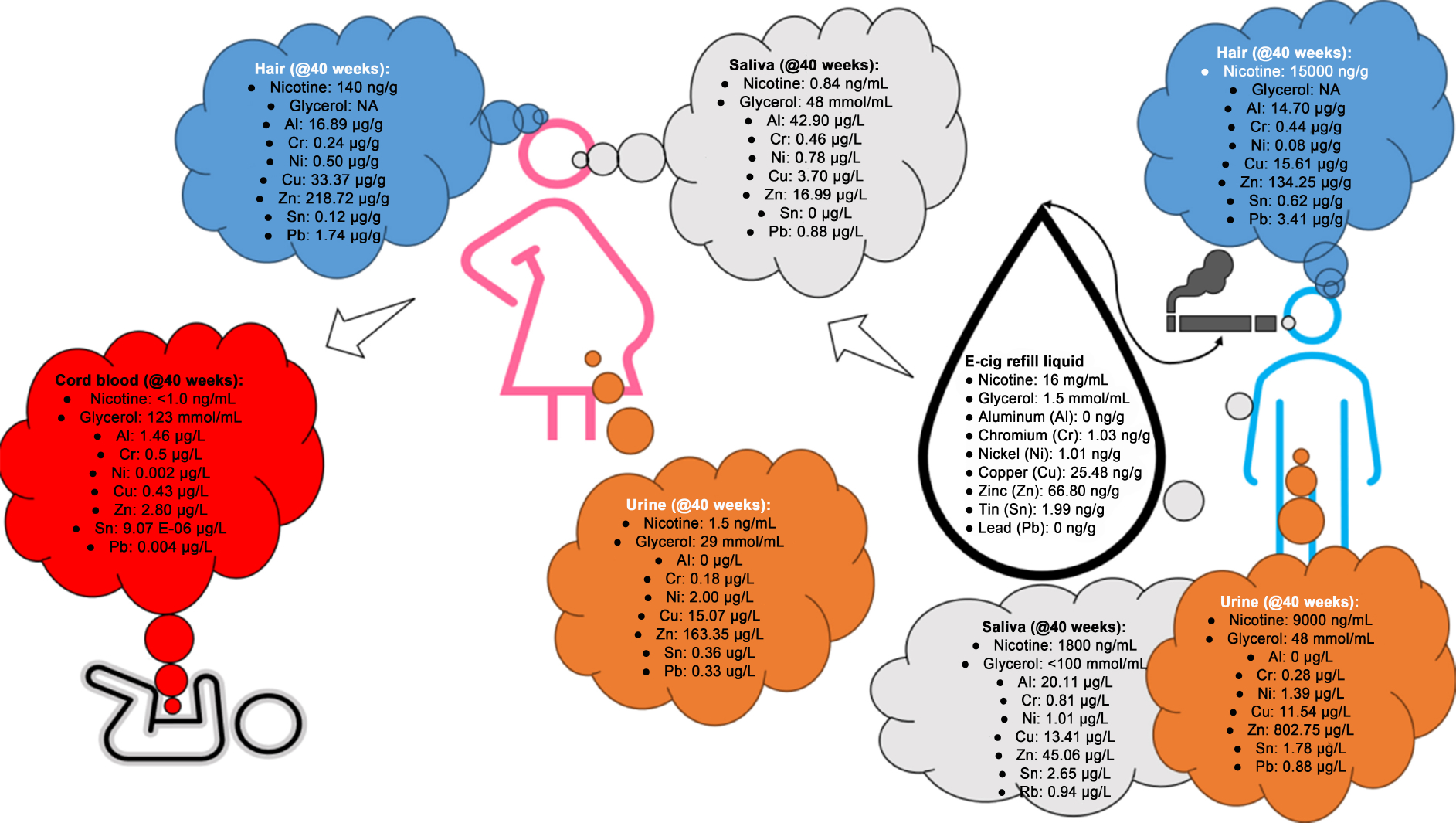
Which chemicals can enter maternal-fetal circulation after ENDS exposure? Select data from the only available human study on passive ENDS exposure in a household by Ballbè *et al.*^[57]^. ENDS: Electronic nicotine delivery systems.

To date, there are no population-level studies on ENDS use and relevant biological concentrations of markers of exposure. In Figure 2, we depict data from the only human study with data on ENDS-associated chemicals and their concentrations in the active user (father), passive subject (mother), and developing fetus. While other time points are available, we only included data collected at 40 weeks of pregnancy for this review. Apart from glycerol, all identified chemicals (mostly metals, i.e., Al, Cr, Ni, Cu, Zn, Sn, and Pb) had much lower concentrations in the cord blood compared to the biological samples collected from the mother or father. The presence of high levels of glycerol in the cord blood compared to other biological matrices in the adults could be due to the contribution from other sources or an accumulation of the product from the passive vape exposures. Identification of glycerol is not uncommon, even when other harmful and potentially harmful constituents, as classified by the FDA, may be undetected^[93]^. Glycerol is an important component of e-liquid formulations. However, when aerosolized during vaping and one happens to be exposed, such exposures may not be trivial because there may be a resulting disruption to immune homeostasis^[94]^. In terms of the metals measured in this study, the actual e-cigarette device may be a potential source and metals consistently found in cartridges include cadmium, nickel, lead, chromium, and arsenic^[30,90]^.

A recent study revealed an important distinction between ENDS users^[45]^. One group inhaled aerosol into their lungs and absorbed most of the chemicals (lung inhalers), while the other group exhaled partially depleted chemicals (mouth inhalers). Pregnant lung inhalers may have a higher potential to increase passive ENDS exposures for a developing fetus since more of the aerosols will deposit into the airways and then potentially to circulation and ultimately to the placenta. Research in this area is essential as it can characterize (1) different chemicals that reach the bloodstream for the two groups (lung/mouth inhalers); (2) which chemicals do reach the fetus; and (3) the downstream signaling molecules from local adverse effects of such chemicals. Tables 3 and 4 show a quantitative comparison between the metal constituents in size-segregated ENDS aerosol, their respective estimated lung-deposited doses, and the health risks of these substances from a collection of popular disposal ENDS products^[30]^. Despite pulmonary deposit being the only studied, this study offers a cancer risk assessment of two target systems: respiratory and central nervous systems. Studies including other systems would add more information to this attempt to quantitatively assess cancer risk after *in utero* exposure to ENDS, and thus identify potential individual cancers specific to the studied systems and sites.

#### Altered immune response in fetus

While traditional cigarette smoking is much examined for an increased risk for childhood cancer^[84,86,87]^, in the offspring of fathers who smoke at the time of conception and/or during pregnancy, nothing is known about the impact of ENDS use. With the novel chemical mix, ENDS use during pregnancy can induce developmental toxicity such as embryonic oxidative stress, which can have lifelong adverse health effects^[95]^. Following maternal exposure to ENDS products, adult offspring of mice have been reported to have increased neuroinflammation^[62]^. For instance, there were increased IL-6 levels in the brain, and elevated IL-6 is involved in carcinogenesis. In a study of a 33-year-old male, Chua *et al.* noted that vaping products may have interacted with endogenous proteins and indirectly trigger autoimmune pulmonary alveolar proteinosis^[61]^. However, one limitation to better understanding these interactions is that this patient had previously used cannabis and e-cigarettes. Nevertheless, both vapors are being addressed in this review. ENDS-associated chemical exposures can also impair DNA repair and alter the functions of lung proteins^[63]^. Nicotine and its nitrosation product 4-(methylnitrosamine)-1-(3-pyridyl)-1-butanone enhanced mutations and tumorigenic cell transformation in cultured human lung and bladder cells^[63]^.

#### DNA damage

ENDS aerosols can cause DNA damage in multiple organs including lungs, hearts and bladders and these effects may occur through direct or indirect action. The ENDS-associated compound could act as a genotoxic agent or can work in association with a compromised immune system. To highlight the latter point, we focus on hydroquinone (HQ), which has been identified as an ENDS-associated chemical. HQ is a group 3 carcinogen since there is inadequate evidence in humans and limited evidence in experimental animals. Yang *et al.* reported that HQ mediates benzene-induced hematopoietic toxicity and can lead to disorders such as aplastic anemia and leukemia^[96]^. It has been suggested that HQ’s carcinogenic action may be through the exacerbation of a natural disease process^[97]^, since it causes genotoxicity in the bone-marrow cells of animal models. Hence, the presence of HQ and/or its metabolites in the uterine environment can work additively or synergistically with other chemicals and influence an offspring’s future health trajectory^[98]^. It is important to stress that thorough research is needed before flavorings, vitamins, and other additives deemed safe for ingestion are also to be assumed as safe for vaping. For a detailed discussion on how ENDS aerosols can induce mitochondrial toxicity, and DNA fragmentation akin to the pathological pathways triggered by tobacco smoke, see the recent review by Vivarelli *et al.*^[99]^.

#### Epigenetics

There was also dysregulation of 24 genes in the lungs of the offspring related to Wnt signaling, 9 genes related to epigenetics, and 7 genes related to inflammation. At 11 weeks of age, JUUL- and house dust mite (HDM)-exposed offspring exhibited pulmonary inflammation compared to their respective air + HDM controls. Additionally, JUUL + HDM female offspring showed decreased methylation of the promoter region of the Il10ra gene^[59]^. Inhalation of JUUL aerosols during pregnancy may affect the intrauterine environment, impair lung development, and heighten the effects of a cascade of adverse health later in life. Additionally, there is the possibility of epigenetic programming that can influence future lung disease in offspring^[58]^. These cascades of events in the future may be due to ENDS-associated byproducts that create a proinflammatory environment within which the fetus develops^[60]^.

#### Gene expression changes

One clear lesson from traditional tobacco use can be gleaned from genotype studies. A single nucleotide polymorphism of the α5 nicotinic receptor [where residue 398 changes aspartic acid, Asp (D), to asparagine, Asn (N) (rs16969968)] is associated with maternal smoking and has detrimental effects on the offspring’s pulmonary function^[100]^. This SNP, combined with smoking, increases the risk of lung cancer and chronic obstructive pulmonary disease^[101]^. Conversely, pregnant e-cig users with this genotype may increase the risk of such adverse health outcomes in their offspring. Individuals who had *in utero* exposure to e-cig aerosols could be predisposed to developing chronic disease later in life, since e-cig aerosols can lead to dysregulated extracellular matrix remodeling, which is in turn associated with pathological conditions such as fibrosis and cancer^[102–104]^. In sum, excessive production of reactive oxygen and nitrogen species by phagocytes can initiate cancer development ^[105]^. On the other hand, *in utero* exposure to e-cigarette aerosols can lead to altered fetal lung gene expression, and this may contribute to future disease development. For instance, Orzabal *et al.* noted that the top 10 downregulated and upregulated genes of offspring exposed to prenatal e-cig aerosols played regulatory roles in the development of diseases such as lung cancers, chronic obstructive pulmonary disease, and asthma^[69]^. Of note, three genes - *Serpinb3a*, *Serpina3c*, and *Serpine3*, which encode serine protease inhibitors, were upregulated. These genes play an important role in lung homeostasis and their overexpression is associated with inflammation^[106]^. It is little wonder that some researchers have cautioned against ENDS use during pregnancy since it can be detrimental to fetal development, due to increases in measures of oxidative stress, inflammation, and fibrosis^[66]^. In a study of both regulated and unregulated e-cigarettes^[54]^, both ENDS- and marijuana vape-associated compounds were found, and maximum concentrations of benzene, methanol, and ethanol in e-liquid samples were higher than their authorized maximum limits as residual solvents in pharmaceutical products. The authors suggested that the solvents used in e-liquids could be ethyl alcohol, methanol, and other petrogenic hydrocarbons and may compromise the safety of e-cig users, as also reported by Lim and Shin^[55]^. Certainly, adverse health effects can occur given the cumulative exposure (at least nine months *in utero* plus postpartum). It is important to note that for some of these studies, the levels of compounds are very low, and ENDS devices were operated outside of the range of typical use and may have generated more byproducts as a result of thermal degradation. Further studies that use settings within the typical range employed by ENDS users are needed to address this research gap.

Before proceeding to the next section of our discussion, it would be remiss to overlook potential methodological changes in several of the studies presented herein. First, there is the issue of aerosol generation that is relevant to humans. A recent review has identified differences between laboratory tests of vape emissions to provide evidence of potential health risks from exposure and actual human experiences^[107]^. For instance, Yan *et al.* performed their experiment at a high power of up to 200 W, with a low airflow of 1 L/min^[56]^. This may have resulted in overheating conditions, which, in turn, led to increased production of aldehydes, but this is not comparable to human vape users. Likewise, in a study by Cahill *et al.*, mice were subjected to whole-body exposure, with aerosols generated through an airflow pump set at 1–2 L/min^[58]^. This setup potentially exposed the animals to high levels of carbonyls. Similarly, Orzabal *et al.*, utilizing a puff volume of volume 42 mL, 1-second puffs every 20 s at 2.5 L/min, and a power of 60 W, probably created overheating conditions, with corresponding high carbonyl levels that do not mirror the settings typical of human vape users^[69]^. Secondly, there is the issue of exposure source. The only human-relevant exposure study^[57]^ highlighted in the current review detected low levels of metals in biological specimens of household members, in comparison to the active vape user. However, the sources of these metals cannot be attributed to the vape alone and could have been derived from other ambient, dermal, or oral pathways of exposure within and outside of the home environment. Taken together, methodological concerns such as inappropriate airflow, coil overheating, and lack of reproducibility may not mimic human-relevant exposures and may lack a translational effect on the potential harms to non-users such as a developing fetus. Well-designed epidemiological studies in the future will help overcome these methodological challenges and answer the ultimate question of whether the preclinical observations translate to altered immune responses and increase cancer risk in the general population. A recent study reported that there were decreased body weights and lengths of exposed mice after birth^[59]^.

### Marijuana vape-contained carcinogens, their transmission from mother to fetus, and the associated altered fetal immune response

Like for ENDS, we also need to understand the impact of actively and/or passively inhaling marijuana vape aerosols. Tang *et al.* highlight the important role of ultrafine particles^[31]^. Such particles act as carriers of low-molecular-weight species and can influence exposure in two ways. First, the chemically bound particles can help transport these chemicals deep into the user’s respiratory system and ultimately into the circulatory system. Secondly, nonusers can be exposed when these chemically bound particles disperse nonvolatile and semi-volatile species in the indoor environment. Whether through this first or second route, the researchers collected air samples and noted that such samples had about 50% of emitted monoterpenes and less than 40% of released sesquiterpenes and terpene alcohols. Furthermore, a large proportion of ultrafine particles were released when the mixture was heated and remained airborne for at least 3 h, and thus, active and passive marijuana vape users can still be exposed to the associated compounds even after ceasing active use. Certainly, further research on the impacts of these emerging vape products is needed to understand their effects on users and nonusers alike.

#### Harmful carcinogens and their transmission

Wu and O’Shea found that the pyrolysis of vitamin E acetate led to the production of carcinogenic alkenes and benzene^[108]^. The authors state that at high temperatures, there is a huge potential for unexpected chemistries to occur among the individual components of a vape mixture. Using a combination of analytical, theoretical, and experimental approaches, Wu and O’Shea identified compounds such as vitamin E acetate, ketene, 2,3,5,6-tetramethylcyclohexa-2,5-diene-1,4-dione, (duroquinone) and prist-1-ene, butadiene, propene, ethene, 2-methylprop-1-ene, and 1-methyl-1-alkyl-alkenes, as well as trace amounts of tetrahydrofuran, formaldehyde, and short-chain aliphatic aldehydes. Ketene (ethenone) has also been reported in vaped condensates from cannabinoid acetates and commercial Δ8-THC acetate products^[109]^. A handful of studies have shown disturbing and detrimental effects of acute ketene toxicity, including inflammation^[110]^; however, long-term consequences of exposure to this compound, by itself or in a mixture with other toxic chemicals, remain unknown. Finally, like in ENDS, metals have also been found in marijuana vaping devices, i.e., nickel, chromium, copper, lead, tin, and gold^[111]^. Like for other metals in ENDS cartridges, nickel and chromium components have been detected in the devices used by patients who were diagnosed with EVALI^[111]^, while other devices contained copper, lead, tin, and gold, which all have the potential to thermally degrade and volatilize when heated. Moving forward, for both ENDS and marijuana vaping devices, healthcare centers, state and federal programs such as the National Health and Nutrition Examination Survey (NHANES)^[112]^ or the Alberta Biomonitoring Program^[113]^ can employ similar strategies to provide effective models for population biomonitoring. Of the metals found in marijuana vaping devices (nickel, chromium, copper, lead, tin, gold)^[111]^, all but gold crossed the placenta barrier in an ENDS study on humans^[57]^. However, we found no study that specifically addressed transmission of carcinogens found in marijuana-vaping devices from pregnant mother to the developing fetus.

#### Altered immune response

Cannabidiol has been shown to induce excessive production of reactive oxygen^[114]^, which, as we said earlier, can initiate cancer development^[105]^. It is therefore important to understand the major constituents in marijuana vape and their amounts in vapor or aerosols that get into a user’s lungs by comparing the major constituents in unused vape oil *vs.* its vaporized and aerosolized forms. While terpenes and minor cannabinoids can be produced when vaporizing and aerosolizing the vape oil, some of these have been found at lower levels in the vapor and aerosol than in the vape liquid^[41]^ and some unlabeled chemicals have been detected^[54,105]^. *In utero* exposure to cannabinoids is associated with T cell dysfunction, an impaired immune response to viruses and susceptibility to cancer^[115]^. In one of the first studies to investigate the gas-phase thermal degradants of Δ9-THC, Meehan-Atrash *et al.* evaluated the safety of THC degradants^[74]^. While this has not been shown in humans, cancer studies in experimental animals showed that these individual chemicals have led to cancer upon exposure. For instance, isoprene caused blood-vessel cancer and benign or malignant tumors of the Harderian gland (adenoma or carcinoma) and the lung (alveolar/bronchiolar adenoma or carcinoma) across both male and female mice. Additionally, in both male and female rats, isoprene caused benign or malignant tumors of the mammary gland (fibroadenoma or carcinoma) and kidney (renal-cell adenoma or carcinoma). Benign tumors of the testis (adenoma) have also been reported in male rats.

#### DNA damage and epigenetic changes

There is also the potential for parental cannabis use to trigger epigenetic modifications (such as altered microRNA, DNA methylation, and histone modification profiles) with long-term transgenerational effects^[115]^. Furthermore, cannabis inhalation is novel and differs from cannabis smoking and there is little current data to correctly assess their safety^[116]^; therefore, further research is needed to assess and characterize this constantly evolving field of commercial vaporizers.

### Limitations

This review has several limitations. First, we limited our search to PubMed, which may have caused us to miss potentially relevant studies retrievable in other databases, including the grey literature^[117,118]^. However, PubMed has the strength of capturing a wide range of studies and a good refinement algorithm, when MeSH term options are used in the search^[119]^. Second, our search was restricted to studies published in English, which may not have accounted for studies published in other languages. Third, not all ENDS and marijuana aerosol/vapor papers were considered for analysis, but only those that pointed to developmental effects or suggested developmental or immune consequences. Even within the aspect of the effects on development, we focused on those addressing cancer development pathways during development - implying long-term effects. Therefore, studies that addressed the short-term effects, such as those of marijuana vaping use during pregnancy on the fetus birthweight or neurodevelopment outcomes, are not discussed. Additionally, we acknowledge but do not discuss other sources and potential for transfer of organic contaminants (or their metabolites) to the developing fetus, as this is beyond the scope of the current work. Finally, our search period ended in December 2022. Given the ongoing influx of new studies on this topic, our findings may not capture the most inclusive update on the topic. However, when considering the 20-year scope of our search, extending it into 2023 at the time of the writing of this review would not have added a high volume of relevant studies to include.

### Thoughts for future directions

In sum, *in utero* exposure to parent or biotransformed ENDS- or marijuana vape-associated chemicals can occur in vital organs such as lungs, kidneys, brain, bladder, and heart. The byproducts can be further metabolized into DNA-damaging agents and increase the risk of tumorigenesis. Such altered intrauterine environment can impair fetal development and heighten the effects of a cascade of future adverse health events, including cancer. However, to date, studies directly assessing related cancer risk after *in utero* exposure in human fetuses are nonexistent. Recent studies showed that exposure to thirdhand smoke exposures from traditional cigarette use can alter body weight and immunity, and lead to lung cancer development in mice^[120]^. One key factor is genetic susceptibility to various forms of cancer, as has been demonstrated in different mouse strains^[121]^. Since some of these chemicals in traditional cigarette smoke have been reported when marijuana and nicotine vape products are used, the adverse health effects of thirdhand smoke from these emerging products need to be thoroughly understood as well. Future epidemiological studies are needed to examine the effects of passive aerosol exposures from existing and emerging electronic nicotine and marijuana products on vulnerable populations including developing fetuses, infants, children, and those with genetic susceptibility to cancer^[88]^.

## Supplementary Material

Supplementary Material

## Figures and Tables

**Table 1.** Studies included in this narrative review for ENDS

Question	Ref.	Species	Exposure	Relevant results

What are the currently identified carcinogens contained in the ENDS aerosols?	Holt *et al.*^[54]^	NA	N/A. 241 e-liquids were submitted for analysis by individuals, purchased directly from manufacturers, or purchased from local retailers	A total of 350 chemicals including nicotine, caffeine, menthol, and ethanol
	Lim and Shin^[55]^	NA	NA. Authors wanted to detect VOCs in refill liquids and cartridges of e-cigs	A total of 14 VOCs were detected including benzene, toluene, ethylbenzene, m-xylene, p-xylene, and o-xylene
	Yan *et al.*^[56]^	NA	Aerosols from two open-system devices were characterized with Orbitrap mass spectrometer	Positive identification of over 30 compounds including nicotine and its oxide compounds, newly formed nicotine adducts with propylene glycol, ethyl maltol, tributylamine, and dibutylamine, as well as some aliphatic and aromatic oxygen-containing compounds which are yet to be determined
Which ones of them are known to cross the placental barrier, and at what proportion compared to their respective concentrations in the mother's blood?	Ballbe *et al.*^[57]^	Human	Passive exposures to daily e-cigarette use from a first-generation device from father	Glycerol and metals (Al, Cr, Ni, Cu, Zn, Sn, and Pb) were in the cord blood
Are these carcinogens associated with altered immune response and (future) risk of cancer in the offspring?	Cahill *et al.*^[58]^	BALB/c mice	Active cinnamon-flavored e-cig aerosols from mother	Altered lung structure and function and sex-specific molecular signatures during lung alveologenesis. Genes associated with Wnt signaling were dysregulated
	Cahill *et al.*^[59]^	BALB/c mice	Active mint-flavored JUUL aerosols from mother	Decreased body weights and lengths after birth. There was also dysregulation of 24 genes in the lungs of the offspring related to Wnt signaling, 9 genes related to epigenetics, and 7 genes related to inflammation. At 11 weeks of age, JUUL + HDM-exposed offspring exhibited pulmonary inflammation compared to their respective air + HDM controls. Additionally, JUUL + HDM female offspring showed decreased methylation of the promoter region of the Il10ra gene
	Chen *et al.*^[60]^	Female BALB/c mice	E-cigarette liquid without nicotine and e-cigarette liquid containing 18 mg/mL of nicotine	TNF-a protein levels were increased in the electronic cigarette groups (compared to controls), and IL-1b was suppressed in adult offspring. Additionally, methylation increased in the e-cigarette liquid without nicotine (~3X) and e-cigarette liquid containing 18 mg/mL of nicotine (~2X)
	Chua *et al.*^[61]^	33-year-old male	Had previously used cannabis and e-cigarettes	Serum had anti-granulocyte-macrophage colony-stimulating factor antibodies were present. Vitamin E (but not VE acetate) was detected in his bronchoalveolar lavage fluid
	Church *et al.*^[62]^	Female CD-1 mice	propylene glycol and vegetable glycerol, or to PG/VG with 16 mg/mL of nicotine	Reduction in IL-4 and IFN-gamma in the diencephalon. There were also lower levels of hippocampal IFN gamma in females only. Additionally, e-cigarette exposure without nicotine resulted in a 2-fold increase of IL-6 in the cerebellum
	Lee et al.^[63]^	FVB/N male mice and human BEAS-2B and UROtsa	10 mg/mL of e-cig aerosols	E-cig aerosols can cause DNA damage in lungs, hearts, and bladders of mice. E-cigs exposures can also impair DNA-repair and alter the functions of lung proteins. Nicotine and its nitrosation product 4-(methylnitrosamine)-1-(3-pyridyl)-1-butanone enhanced mutations and tumorigenic cell transformation in cultured human lung and bladder cells
	Hickman *et al.*^[64]^	Neutrophils from the blood of healthy human subjects	“Cinnamaldehyde, ethyl vanillin, benzaldehyde, benzaldehyde PG acetal, and isoamyl acetate"	Cinnamaldehyde and ethyl vanillin exposures led to decreased oxidative burst. Benzaldehyde and benzaldehyde propylene glycol acetal impaired phagocytosis
	Landmesser *et al.*^[65]^	Humans	25 healthy adult males [5 regular smokers, 10 regular vapers (vaping labeled e-liquid at 10 W), and 10 regular vapers (vaping labeled e-liquid at 18 W)]	Degradation products formaldehyde acetaldehyde and acrolein were formed from the precursors' propylene glycol and glycerol in e-liquid during vaping
	Li *et al.*^[66]^	BALB/c mice	Mice were exposed to air or tobacco cigarette smoke for 6 weeks prior to mating, during gestation and lactation. Then, a subset received e-cigarettes with nicotine after mating until pups weaned. Lastly, another group was exposed to e-cigs with or without nicotine for 6 weeks prior to mating until pups weaned	In comparison to dams with continuous cigarette smoke exposures, replacement of traditional cigarette smoke with e-cig resulted in less adverse impacts on the offspring's renal development and albuminuria. However, continuous e-cig exposure during pregnancy resulted in increased kidney markers of oxidative stress, renal inflammation, and fibrosis in the adult offspring
	Li *et al.*^[67]^	BALB/c mice	Mice were exposed to e-cigs with or without nicotine for 6 weeks prior to mating until pups weaned	E-cig (without nicotine) exposure during pregnancy led to metabolic changes and liver damage in offspring. On the other hand, e-cigs with nicotine led to induced hepatic steatosis but not liver damage in the adult offspring
	Noel *et al.*^[68]^	BALB/c mice	Mice were exposed to 36 mg/mL of nicotine cinnamon-flavored e-cig aerosols for 14–31 days [for either 12 days before mating plus during gestation (preconception groups) or only during gestation (prenatal groups)]	There was an e-cig immunosuppressive effect among the mice exposed to e-cigs during preconception and prenatal exposures. The exposed offspring had decreased birth weights and lengths, and in the preconception group, 7 inflammation-related genes were downregulated. Additionally, 75 genes involved in Wnt signaling were downregulated
	Orzabal *et al.*^[69]^	Sprague Dawley rats	Mice were exposed to aerosols from e-cigs with and without nicotine (e-cig base had 80:20 propylene glycol and glycerol	There were 984 genes that were downregulated and 2,322 genes that were upregulated among offspring exposed to e-cigs compared to unexposed offspring and these differentially expressed genes mapped to 159 disrupted cellular pathways
	Rickard *et al.*^[70]^	HepG2 Cells	Cells were exposed to isoamyl acetate, vanillin, ethyl vanillin, ethyl maltol, L-menthol, and trans-cinnamaldehyde	Vanillin, ethyl vanillin, ethyl maltol, and L-menthol exposure led to the decreased viability of HepG2 cells (>30% decrease in cell viability). On the other hand, PG/VG may not decrease viability on their own but may inflict damage when contained in a mixture with various flavoring chemicals
	Wang *et al.*^[71]^	CD-1 mice	E-cig aerosols (50% PG, 50% VG, and 16% nicotine)	Adult offspring of mice exposed to in-utero e-cig had an increased abundance of lymphoid enhancer-binding factor 1, fibronectin, and E-cadherin. On the other hand, altered E-cadherin and peroxisome proliferator-activated receptor γ levels were observed only in males exposed to e-cig aerosols with nicotine. Then, lipogenic and myogenic mRNAs were dysregulated in adult offspring in a sex-dependent manner. PAI-1, an extracellular matrix regulator, was significantly increased in females exposed prenatally to e-cig aerosols with nicotine and in males exposed to e-cig aerosols in comparison to control animals. Matrix metalloproteinase 9, a downstream target of PAI-1, was downregulated in both sexes exposed to e-cig aerosols with nicotine during pregnancy

ENDS: Electronic nicotine delivery systems; VOCs: volatile organic compounds; BALB/c: BALB/c laboratory mouse strain; JUUL: JUUL e-cigarette brand; HDM: house dust mite; TNF: tumor necrosis factor; IL: interleukin; CD-1: CD-1 laboratory mouse strain; PG: propylene glycol; VG: vegetable glycerin; IFN: interferon; FVB/N: FVB/N laboratory mouse strain; BEAS-2B: BEAS-2B human airway epithelial cell line; UROtsa: a simian virus 40 (SV40) immortalized human urothelium cell line; PAI-1: plasminogen activator inhibitor-1

**Table 2.** Studies included in this narrative review for marijuana vaping

Question	Ref.	Species	Exposure	Relevant results

What are the currently identified carcinogens contained in marijuana vapor?	Tang *et at.*^[31]^	NA	Mixtures of different proportions of 12 vaporizable cannabis concentrates (terpenoids and high molecular weight compounds) were heated at 100–500 °C in a room-sized chamber to simulate emissions	Acrolein (1.3–3.9 μg⋅m^−3^) and methacrolein (2.0 μg⋅m^−3^), along with nine other degradation byproducts, were quantified, and a large amount of ultrafine particles were released when the mixture was heated and remained airborne for at least 3 h
	Canchola *et al.*^[72]^	NA	Vitamin E acetate vaping emissions (temperature ranging from 176 to 356 °C)	DL-alpha tocopherol acetate, 1-Pristene, 1-Heptene, 2,6- dimethyl, DL-alpha tocopherol, Duroquinone, 1-Dodecanol, 3,7,11-trimethyl, 1-Undecene, 4-methyl, 1-Heptene, 2- methyl, Durohydroquinone, 1-Decene, 4 methyl, 2,6,10- Trimethylundeca-1,3-diene, 3,7,11,15-Tetramethyl-2- hexadecen-1-ol (Phytol), Dodecane,2,6,10-trimethyl, 1,4- Benzenediol, 2,3,5-trimethyl, 2,6-Dimethyl-1,6-heptadiene, 1-Octene,3,7-dimethyl, and 1-Octene,3-methyl found at different temperatures
	Guo *et al.*^[41]^	NA	N/A. Twelve cannabis vape cartridges were obtained through the California state surveillance program and major constituents in vape oil liquid, vape oil vapor, and vape oil aerosol phases were tested	Identified more than 100 terpenes and natural extracts, 19 cannabinoids, and other potential toxic additives such as Vitamin E Acetate, Polyethylene Glycols, and Medium Chain Triglycerides
	LeBouf *et al.*^[73]^	NA	NA. vitamin E acetate, vitamin E, coconut, and MCT oil	Alcohols, aldehydes, ketones, and saturated and unsaturated hydrocarbons were identified and were due to volatilization, thermal degradation, or reactions (oxidation or rearrangements)
	Li *et al.*^[43]^	NA	NA. To assess carbonyls, organic acids, cannabinoids, and terpenes in the vaping aerosol of pure VEA, purified THC oil, and an equal volume mixture of VEA and THC oil at various coil temperatures (100–300 °C)	The degradation of VEA and cannabinoids occurred via radical oxidation and direct thermal decomposition pathways. The authors observed that THC oil tended to aerosolize and degrade more compared to VEA at a given temperature. Toxic carbonyls such as formaldehyde, 4- methylpentanal, glyoxal, or diacetyl and its isomers increased in VEA e-liquid when normalized to particle mass
	Meehan- Atrash *et al.*^[74]^	NA	NA. To evaluate the safety of vaporizing concentrated cannabis extracts as a function of gas-phase reaction products	Users may be exposed to degradants such as isoprene (3.0 × 10^-2^ - 6.0 × 10^0^ μg), methacrolein (5.6 × 10^−3^ - 1.9 × 10 ^−1^ μg), benzene (9.9 × 10 ^−1^ - 3.6 × 10^1^ ng), and methyl vinyl ketone when using cartridge vaporizers at 3.2 V, 4.0 V, and 4.8 V, and higher quantities when using dabbing Additionally, the study estimated cancer and noncancer risks associated with exposure to these degradants in comparison with dabbing
Which ones of them are known to cross the placental barrier, and at what proportion compared to their respective concentrations in the mother's blood?	No study identified			
Are these carcinogens associated with (future) risk of cancer in the offspring?	Canchola *et al.*^[75]^	Human bronchial epithelial cells (BEAS-2B)	Duroquinone in vitamin E acetate vaping emissions	Generated ROS and induced oxidative stress-associated gene expression, including upregulation of NQO1 and HMOX-1
	Chen *et al.*^[76]^	p53- deficient female and male mice	10% (w/w) vitamin E (dl-a-tocopherol acetate)	Fetal skin had increased DNA oxidation in homozygous or heterozygous p53-deficient fetuses and enhanced tumorigenesis in such genotypes
	Jiang *et al*^[77]^	Human bronchial epithelial cells (BEAS-2B)	Vape liquid diluents were purchased from commercial sources	The presence of thermally transformed toxic byproducts (such as carbonyls, esters, alkyl alcohols, and quinones) inhibited cell proliferation and enhanced cytotoxicity

DL-alpha-tocopherol: synthetic form of alpha-tocopherol, where D repersent the natural form and DL the synthetic one; MCT: medium chain triglyceride; VEA: vitamin E acetate; THC: tetrahydrocannabinol; ROS: reactive oxygen species; NQO1: NAD(P)H quinone dehydrogenase 1, [NAD(P)H: Nicotinamide adenine dinucleotide phosphate]; HMOX-1: heme oxygenase I.

**Table 3.** Estimated lung-deposited doses and cancer and noncancer risks of ENDS aerosol generated from disposable e-cigarettes, by Air Bar - Watermelon Ice^[30]^

	Estimated dose		Cancer risk assessment			
			
Chemical	LADD (g/kg/Day)	ADD (g/kg/Day)	CSF (g/kg/Day)^−1^	Cancer risk	Target system	HI

Chromium	1.96 × 10^−3^	4.58 × 10^−3^	^a^5.1 × 10^−1^	**1.0 × 10^−3^**	Respiratory	1.14 (Respiratory)
Nickel	1.68 × 10^−3^	3.91 × 10^−3^	^a^9.1 × 10^−4^	**1.5 × 10^−6^**	Respiratory	
Manganese	4.60 × 10^−4^	1.07 × 10^−3^		-	CNS	0.11 (CNS)
Lead	5.70 × 10^−4^	1.32 × 10^−3^	^a^4.2 × 10^−5^	2.4 × 10^−8^	CNS	
Aluminum	1.02 × 10^−2^	2.38 × 10^−2^	^b^-	-	-	
Zinc	1.07 × 10^−2^	2.50 × 10^−2^	^b^-	-	-	

a Information extracted from CalEPA;

b No established CSF or RfC; Bold: exceeds acceptance risk (i.e., cancer risk > 1.0 × 10 ^−6^; HQ > 1.0; HI > 1.0). ENDS: Electronic nicotine delivery systems; LADD: lifetime average daily dose; ADD: average daily dose; CSF: cancer slope factor; HI: hazard index; CNS: central nervous system; HQ: hazard quotient.

**Table 4.** Estimated lung-deposited doses and cancer risks of ENDS aerosol generated from disposable e-cigarettes, by Puff Bar-Grape^[30]^

	Estimated dose		Cancer Risk assessment			
			
Chemical	LADD (g/kg/Day)	ADD (g/kg/Day)	CSF (g/kg/Day)^−1^	Cancer risk	Target system	HI

Chromium	7.60 × 10^−4^	1.78 × 10^−3^	^a^5.1 × 10^−1^	**3.9 × 10^−4^**	Respiratory	0.57 (Respiratory)
Nickel	8.70 × 10^−4^	2.04 × 10^−3^	^a^9.1 × 10^−4^	8.0 × 10^−7^	Respiratory	
Manganese	2.20 × 10^−4^	5.10 × 10^−4^	-	-	CNS	< 0.05 (CNS)
Lead	9.00 × 10^−5^	2.10 × 10^−4^	^a^4.2 × 10^−5^	3.8 × 10^−9^	CNS	
Aluminum	3.87 × 10^−3^	9.03 × 10^−3^	^b^-	-	-	-
Zinc	6.13 × 10^−3^	1.43 × 10^−2^	^b^-	-	-	-

a Information extracted from CalEPA;

b No established CSF or RfC. Bold: exceeds acceptance risk (i.e., cancer risk > 1.0 × 10^−6^; HQ > 1.0; HI > 1.0). ENDS: Electronic nicotine delivery systems; LADD: lifetime average daily dose; ADD: average daily dose; CSF: cancer slope factor; HI: hazard index; CNS: central nervous system; HQ: hazard quotient.

## Data Availability

The complete PubMed search strategy used for this review is provided in the Supplementary Materials.
