# Supplementary material for: Nicotine or marijuana vaping exposure during pregnancy and altered immune responses in offspring
Source: J Environ Expo Assess. Author manuscript; Available in PMC 2024 Jun 5. (PMC11152453; doi:10.20517/jeea.2024.03)
Supplement: Supplementary Material [file NIHMS1991512-supplement-Supplementary_Material.pdf]

Ndeke *et al. J Environ Expo Assess* 2024;3:11
DOI: 10.20517/jeea.2024.03

Journal of Environmental Exposure Assessment

Nicotine or marijuana vaping exposure during pregnancy and altered immune responses in offspring

Jonas M. Ndeke[1], James E. Klaunig[2], Sarah Commodore[2]

[1]Department of Epidemiology and Biostatistics, Indiana University School of Public Health, Bloomington, IN 47405, USA.
[2]Department of Environmental and Occupational Health, Indiana University School of Public Health, Bloomington, IN 47408, USA.

Correspondence to: Jonas M. Ndeke, Department of Epidemiology and Biostatistics, Indiana University School of Public Health, 1025 E. 7th Street, Room 029, Bloomington, IN 47405, USA. E-mail: jndeke@iu.edu

Search Terms Used in PubMed for the Narrative Review on
Electronic Nicotine Delivery Systems Use in Pregnancy and Cancer Risk in Offspring
Search last performed: 1/31/2023, Filtered by 2003-12/31/2022 Period.

1. **Search #1** (listed #1 in PubMed): All compounds > **0 results**

(“Nicotine”[Mesh] OR “nicotine”[tiab] OR “chromium”[MeSH] OR “chromium”[tiab] OR “cadmium”[MeSH] OR “cadmium”[tiab] OR “lead”[MeSH] OR “lead”[tiab] OR “nickel”[MeSH] OR “nickel”[tiab] OR “Manganese”[Mesh] OR “Manganese”[tiab] OR “acrolein”[MeSH Terms] OR “acrolein”[tiab] OR “acetaldehyde”[MeSH Terms] OR “acetaldehyde”[tiab] OR “Benzaldehyde*”[tiab] OR “Formaldehyde”[tiab] OR “propylene glycol”[MeSH Terms] OR “propylene glycol”[tiab] OR “carcinogenic tobacco-specific nitrosamines”[tiab] OR “dronabinol”[MeSH Terms] OR “tetrahydrocannabinol”[tiab] OR “benzene”[MeSH Terms] OR “benzene”[tiab] OR “vitamin e”[MeSH Terms] OR “vitamin e”[tiab]) AND (“acetates”[MeSH Terms] OR “acetate”[tiab] OR “methacrylaldehyde”[All Fields] OR “methacrolein”[tiab])
AND
(“Electronic Nicotine Delivery Systems”[Mesh] OR “Electronic Nicotine Delivery Systems”[tiab] OR “marijuana vap*”[tiab] OR “vaping”[mesh] OR “vape”[tiab] OR “vaping”[tiab] OR “vapor”[tiab] OR “e-vapor”[tiab] OR “evapor”[tiab] OR “e vapor”[tiab])
AND
(“Pregnancy”[MeSH] OR “pregnancy”[tiab])

2. **Search #2** (listed #2 in PubMed):: All compounds and ENDS, without pregnancy > **194 results**

(“Nicotine”[Mesh] OR “nicotine”[tiab] OR “chromium”[MeSH] OR “chromium”[tiab] OR “cadmium”[MeSH] OR “cadmium”[tiab] OR “lead”[MeSH] OR “lead”[tiab] OR “nickel”[MeSH] OR “nickel”[tiab] OR “Manganese”[Mesh] OR “Manganese”[tiab] OR “acrolein”[MeSH Terms] OR “acrolein”[tiab] OR “acetaldehyde”[MeSH Terms] OR “acetaldehyde”[tiab] OR “Benzaldehyde*”[tiab] OR “Formaldehyde”[tiab] OR “propylene

glycol”[MeSH Terms] OR “propylene glycol”[tiab] OR “carcinogenic tobacco-specific nitrosamines”[tiab] OR “dronabinol”[MeSH Terms] OR “tetrahydrocannabinol”[tiab] OR “benzene”[MeSH Terms] OR “benzene”[tiab] OR “vitamin e”[MeSH Terms] OR “vitamin e”[tiab]) AND (“acetates"[MeSH Terms] OR “acetate”[tiab] OR “methacrylaldehyde”[All Fields] OR “methacrolein”[tiab])
AND
(“Electronic Nicotine Delivery Systems”[Mesh] OR “Electronic Nicotine Delivery Systems”[tiab] OR “marijuana vap*”[tiab] OR “vaping”[mesh] OR “vape”[tiab] OR “vaping”[tiab] OR “vapor”[tiab] OR “e-vapor”[tiab] OR “evapor”[tiab] OR “e vapor”[tiab])

3. **Search #3** (listed #3 in PubMed):: Three parts, with e-cig compounds only **> 179 results**

(“Nicotine”[Mesh] OR “nicotine”[tiab] OR “chromium”[MeSH] OR “chromium”[tiab] OR “cadmium”[MeSH] OR “cadmium”[tiab] OR “lead”[MeSH] OR “lead”[tiab] OR “nickel”[MeSH] OR “nickel”[tiab] OR “Manganese"[Mesh] OR “Manganese”[tiab] OR “acrolein”[MeSH Terms] OR “acrolein”[tiab] OR “acetaldehyde”[MeSH Terms] OR “acetaldehyde”[tiab] OR “Benzaldehyde*”[tiab] OR “Formaldehyde”[tiab] OR “propylene glycol”[MeSH Terms] OR “propylene glycol”[tiab] OR “carcinogenic tobacco-specific nitrosamines”[tiab])
AND
(“Electronic Nicotine Delivery Systems”[Mesh] OR “Electronic Nicotine Delivery Systems”[tiab] OR “marijuana vap*”[tiab] OR “vaping”[mesh] OR “vape”[tiab] OR “vaping”[tiab] OR “vapor”[tiab] OR “e-vapor”[tiab] OR “evapor”[tiab] OR “e vapor”[tiab])
AND
(“Pregnancy”[MeSH] OR “pregnancy”[tiab])

4. **Search #4** (listed #4 in PubMed):: Three parts, with marijuana vaping compounds only **> 0 results**

(“dronabinol”[MeSH Terms] OR “tetrahydrocannabinol”[tiab] OR "benzene”[MeSH Terms] OR “benzene”[tiab] OR “vitamin e”[MeSH Terms] OR “vitamin e”[tiab]) AND (“acetates”[MeSH Terms] OR “acetate”[tiab] OR “methacrylaldehyde”[All Fields] OR “methacrolein”[tiab])
AND
(“Electronic Nicotine Delivery Systems”[Mesh] OR “Electronic Nicotine Delivery Systems”[tiab] OR “marijuana vap*”[tiab] OR “vaping”[mesh] OR “vape”[tiab] OR “vaping”[tiab] OR “vapor”[tiab] OR “e-vapor”[tiab] OR “evapor”[tiab] OR “e vapor”[tiab])
AND
(“Pregnancy”[mesh] OR “pregnancy”[tiab])

5. **Search #5** (listed #5 in PubMed):: Two parts, without pregnancy and with marijuana vaping compounds only **> 129 results**

(“dronabinol”[MeSH Terms] OR “tetrahydrocannabinol”[tiab] OR “benzene”[MeSH Terms] OR “benzene”[tiab] OR “vitamin e”[MeSH Terms] OR “vitamin e”[tiab]) AND (“acetates”[MeSH Terms] OR “acetate”[tiab] OR “methacrylaldehyde”[All Fields] OR “methacrolein”[tiab])
AND
("Electronic Nicotine Delivery Systems"[Mesh] OR “Electronic Nicotine Delivery Systems”[tiab] OR “marijuana vap*”[tiab] OR “vaping”[mesh] OR “vape”[tiab] OR “vaping”[tiab] OR “vapor”[tiab] OR “e-vapor”[tiab] OR “evapor”[tiab] OR “e vapor”[tiab])

6. **Search #6** (listed #6 in PubMed):: Two parts, with pregnancy and ENDS (excluding compounds) **> 391 results**

(“Electronic Nicotine Delivery Systems”[Mesh] OR “Electronic Nicotine Delivery Systems”[tiab] OR “marijuana vap*”[tiab] OR “vaping”[mesh] OR “vape”[tiab] OR “vaping”[tiab] OR “vapor”[tiab] OR “e-vapor”[tiab] OR “evapor”[tiab] OR “e vapor”[tiab])
AND
(“Pregnancy”[mesh] OR “pregnancy”[tiab])

7. **Search #7** (listed #7 in PubMed):: Two parts, with pregnancy and marijuana vaping only (excluding compounds and other ENDS) **> 0 results**

("marijuana vap*"[tiab])
AND
("Pregnancy"[mesh] OR "pregnancy"[tiab])

8. **Search #8** (listed #8 in PubMed):: two parts, with marijuana OR cannabis and pregnancy only > **829 results**

("marijuana vaping"[tiab] OR "marijuana e-vapor"[tiab] OR "marijuana evapor"[tiab] OR "marijuana vapor"[tiab] OR "marijuana vape"[tiab] OR "cannabis"[tiab])
AND
("Pregnancy"[mesh] OR "pregnancy"[tiab])

9. **Search #9** (listed #9 in PubMed): Particular searches

"tobacco-specific nitrosamines" AND ENDS AND pregnancy > **1 result**

10. **Search #10** (listed #10 in PubMed): excluding ENDS **> 281 results**

("Nicotine"[Mesh] OR "nicotine"[tiab] OR "chromium"[MeSH] OR "chromium"[tiab] OR "cadmium"[MeSH] OR "cadmium"[tiab] OR "lead"[MeSH] OR "lead"[tiab] OR "nickel"[MeSH] OR "nickel"[tiab] OR "Manganese"[Mesh] OR "Manganese"[tiab] OR "acrolein"[MeSH Terms] OR "acrolein"[tiab] OR "acetaldehyde"[MeSH Terms] OR "acetaldehyde"[tiab] OR "Benzaldehyde*"[tiab] OR "Formaldehyde"[tiab] OR "propylene glycol"[MeSH Terms] OR "propylene glycol"[tiab] OR "carcinogenic tobacco-specific nitrosamines"[tiab] OR "dronabinol"[MeSH Terms] OR "tetrahydrocannabinol"[tiab] OR "benzene"[MeSH Terms] OR "benzene"[tiab] OR "vitamin e"[MeSH Terms] OR "vitamin e"[tiab]) AND ("acetates"[MeSH Terms] OR "acetate"[tiab] OR "methacrylaldehyde"[All Fields] OR "methacrolein"[tiab])

AND
(“Pregnancy”[mesh] OR “pregnancy”[tiab])

Total results before Marijuana compound search (including duplicates): 2004

Searches for marijuana compounds (including flavors) - last searched on 1/31/2023.

11. Search #11 (listed #11 in PubMed): (“caryophyllene”[All Fields] OR Caryophyllene[tiab]) AND (“Pregnancy”[mesh] OR “pregnancy”[tiab]) > **5 results**
12. Search #12 (listed #12 in PubMed): “Alpha-Bisabolol” [tiab] AND (“Pregnancy”[mesh] OR “pregnancy”[tiab]) > **2 results**
13. Search #13 (listed #13 in PubMed): “linalool”[All Fields] OR “Linalool”[tiab] AND (“Pregnancy”[mesh] OR “pregnancy”[tiab]) > **5 results**
14. Search #14 (listed #14 in PubMed): (“humulene”All Fields] OR “Alpha-Humulene”[tiab]) AND (“humulene”[All Fields] OR “Humulene”[tiab]) AND (“Pregnancy”[mesh] OR “pregnancy”[tiab]) > **2 results**
15. Search #15 (listed #15 in PubMed): (“limonene”[MeSH Terms] OR D-limonene[tiab]) AND (“Pregnancy”[mesh] OR “pregnancy”[tiab]) > **5 results**
16. Search #16 (listed #16 in PubMed): (“phytol”[MeSH Terms] OR Phytol[tiab]) AND (“Pregnancy”[mesh] OR “pregnancy”[tiab]) > **161 results**
17. Search #17 (listed #17 in PubMed): (Caryophyllene oxide [tiab]) AND (“Pregnancy”[mesh] OR “pregnancy”[tiab]) > **0 results**
18. Search #18 (listed #18 in PubMed): (Eudesma-3,7(11)-diene [tiab] OR Selina-3,7(11)-diene [tiab]) AND (“Pregnancy”[mesh] OR “pregnancy”[tiab]) > **0 results**
19. Search #19 (listed #19 in PubMed): (“fenchol”[All Fields] OR Fenchol[tiab]) AND (“Pregnancy”[mesh] OR “pregnancy”[tiab]) >**0 results**
20. Search #20 (listed #20 in PubMed): (“nerolidol”[All Fields] OR Nerolidol[tiab]) AND (“nerolidol”[All Fields] OR -Nerolidol[tiab]) AND (“Pregnancy”[mesh] OR “pregnancy”[tiab]) > **1 results**
21. Search #21 (listed #21 in PubMed): (“stanol”[MeSH Terms] OR Squalene[tiab]) AND (“Pregnancy”[mesh] OR “pregnancy”[tiab]) > **35 results**

22. Search #22 (listed #22 in PubMed): "Gamma-Selinene" [tiab] AND ("Pregnancy"[mesh] OR "pregnancy"[tiab]) > **0 results**

23. Search #23 (listed #23 in PubMed): ("beta-myrcene"[All Fields] OR Beta-Myrcene[tiab]) AND ("Pregnancy"[mesh] OR "pregnancy"[tiab]) > **0 results**

24. Search #24 (listed #24 in PubMed): "Terpinolene"[tiab] AND ("Pregnancy"[mesh] OR "pregnancy"[tiab]) > **0 results**

25. Search #25 (listed #25 in PubMed): "beta-pinene"[All Fields] OR Beta-Pinene[tiab] AND ("Pregnancy"[mesh] OR "pregnancy"[tiab]) > **2 results**

26. Search #26 (listed #26 in PubMed): "Alpha-Selinene"[tiab] AND ("Pregnancy"[mesh] OR "pregnancy"[tiab]) > **0 results**

27. Search #27 (listed #27 in PubMed): "Alpha-Eudesmol" [tiab] AND ("Pregnancy"[mesh] OR "pregnancy"[tiab]) > **0 results**

28. Search #28 (listed #28 in PubMed): "Guaiol" [tiab] AND ("Pregnancy"[mesh] OR "pregnancy"[tiab]) > **1 results**

29. Search #29 (listed #29 in PubMed): ("valencene"[All Fields] OR Valencene[tiab]) AND ("Pregnancy"[mesh] OR "pregnancy"[tiab]) > **0 results**

30. Search #30 (listed #30 in PubMed): "Beta-Maaliene"[tiab] AND ("Pregnancy"[mesh] OR "pregnancy"[tiab]) > **0 results**

31. Search #31 (listed #31 in PubMed): (Copaene[All Fields] OR "copaene"[tiab]) AND ("Pregnancy"[mesh] OR "pregnancy"[tiab]) > **0 results**

32. Search #32 (listed #32 in PubMed): ("isoborneol"[All Fields] OR Borneol[tiab] OR "Endo-borneol" [tiab]) AND ("Pregnancy"[mesh] OR "pregnancy"[tiab]) > **1 results**

33. Search #33 (listed #33 in PubMed): "Neophytadiene" [tiab] AND ("Pregnancy"[mesh] OR "pregnancy"[tiab]) > **0 results**

34. Search #34 (listed #34 in PubMed): "Bulnesol" [tiab] AND ("Pregnancy"[mesh] OR "pregnancy"[tiab]) > **0 results**

35. Search #35 (listed #35 in PubMed): "m-Camphorene" [tiab] AND ("Pregnancy"[mesh] OR "pregnancy"[tiab]) > **0 results**

36. Search #36 (listed #36 in PubMed): ("Delta-Guaiene"[tiab] OR ("alpha-bulnesene"[All Fields] OR "Alpha-Bulnesene"[tiab])) AND ("Pregnancy"[mesh] OR "pregnancy"[tiab]) > **0 results**

37. Search #37 (listed #37 in PubMed): ("gamma-eudesmol"[All Fields] OR "Gamma-Eudesmol"[tiab]) AND ("Pregnancy"[mesh] OR "pregnancy"[tiab]) **> 0 results**
38. Search #38 (listed #38 in PubMed): "Junipercamphor" [tiab] AND ("Pregnancy"[mesh] OR "pregnancy"[tiab]) **> 0 results**
39. Search #39 (listed #39 in PubMed): ("alloaromadendrene"[All Fields] OR Alloaromadendrene[tiab]) AND ("Pregnancy"[mesh] OR "pregnancy"[tiab]) **> 0 results**
40. Search #40 (listed #40 in PubMed): "Beta-Cadinene"[tiab] AND ("Pregnancy"[mesh] OR "pregnancy"[tiab]) **> 0 results**
41. Search #41 (listed #41 in PubMed): ("isoledene"[All Fields] OR Isoledene[tiab]) AND ("Pregnancy"[mesh] OR "pregnancy"[tiab]) **> 0 results**
42. Search #42 (listed #42 in PubMed): "2-Pinene"[tiab] AND ("Pregnancy"[mesh] OR "pregnancy"[tiab]) **> 0 results**
43. Search #43 (listed #43 in PubMed): ("3-carene"[All Fields] OR 3-Carene[tiab] OR "Delta-3-carene"[tiab]) AND ("Pregnancy"[mesh] OR "pregnancy"[tiab]) **> 0 results**
44. Search #44 (listed #44 in PubMed): ("isoborneol"[All Fields] OR Isoborneol[tiab]) AND ("Pregnancy"[mesh] OR "pregnancy"[tiab]) **> 0 results**
45. Search #45 (listed #45 in PubMed): "(E)-Beta-Famesene" [tiab] OR "Beta-Famesene"[tiab] AND ("Pregnancy"[mesh] OR "pregnancy"[tiab]) **> 0 results**
46. Search #46 (listed #46 in PubMed): "Aromandendrene"[tiab] AND ("Pregnancy"[mesh] OR "pregnancy"[tiab]) **> 0 results**
47. Search #47 (listed #47 in PubMed): (("alpha-terpineol"[All Fields] OR Alpha-Terpineol[tiab]) OR -Alpha-Terpineol[tiab])) AND ("Pregnancy"[mesh] OR "pregnancy"[tiab]) **> 1 results**

Total results (as of 1/31/2023 and filtered from 1992 to 12/31/2022): **2225** studies (including duplicates)

Combination of searches

48. **Search #48** = #2 + #5 **> 184** (same as Search #2).
